# Evidence Synthesis and Translation for Nutrition Interventions to Combat Micronutrient Deficiencies with Particular Focus on Food Fortification

**DOI:** 10.3390/nu8090555

**Published:** 2016-09-08

**Authors:** Mark Lawrence, Kate Wingrove, Celeste Naude, Solange Durao

**Affiliations:** 1 Institute for Physical Activity and Nutrition, Deakin University, 221 Burwood Highway, Burwood 3125, Australia; k.wingrove@deakin.edu.au; 2 Centre for Evidence-Based Health Care, Faculty of Medicine and Health Sciences, Stellenbosch University, Francie van Zijl Drive, Tygerberg 7505, South Africa; cenaude@sun.ac.za; 3 Cochrane Nutrition, Centre for Evidence-based Health Care, Stellenbosch University and South African Cochrane Centre, South African Medical Research Council, Francie van Zijl Drive, Tygerberg 7505, South Africa; Solange.Durao@mrc.ac.za; 4 South African Cochrane Centre, South African Medical Research Council, Francie van Zijl Drive, Tygerberg 7505, South Africa

**Keywords:** food fortification, micronutrient deficiency, evidence synthesis, policy, systematic review, nutrition-specific, nutrition-sensitive

## Abstract

Over two billion people suffer from micronutrient deficiencies. Food fortification is a prominent nutrition intervention to combat such deficiencies; however, its effectiveness, risks, and ethical implications vary depending on the contexts associated with the deficiency it is addressing and the circumstances with its implementation. The aim of this research was to analyse the profile of nutrition interventions for combating micronutrient deficiency with particular focus on food fortification reported in existing systematic reviews (SRs), guidelines and policy statements, and implementation actions for nutrition. A review of secondary data available from online databases of SRs, guidelines and policy statements, and implementation actions, categorised as either “nutrition-specific interventions” (NSpI) or “nutrition-sensitive interventions” (NSeI), was conducted. Currently, there is evidence available for a diversity of food fortification topics, and there has been much translation into action. Indeed, food fortification and micronutrient supplementation interventions and NSpI more broadly dominate the profile of interventions for which there were SRs, guidelines, and policy statements available. The findings demonstrate that, although there is a rational linear relationship between evidence synthesis and translation in formulating policy and actions to combat micronutrient deficiencies, the various nutrition interventions available to help combat micronutrient deficiencies are not equally represented in the evidence synthesis and translation processes. Effective and safe policies and actions to combat micronutrient deficiencies require decisions to be informed from a body of evidence that consists of evidence from a variety of interventions. Into the future, investment in making available a higher number of SRs, guidelines and policy statements, and actions of NSeI is indicated.

## 1. Introduction

From 19 to 21 November 2014, the Rome Declaration on Nutrition, the outcome document of the Second International Conference on Nutrition (ICN2), noted that over two billion people suffer from micronutrient deficiencies and in particular vitamin A, iodine, iron, and zinc [1]. The ICN2 Framework for Action [2] and the subsequent United Nations General Assembly proclamation on the Decade for Action on Nutrition 2016–2025 [3] have recommended urgent and significant action to combat micronutrient deficiencies. Food fortification, the addition of a nutrient(s) to a food to prevent or correct a demonstrated nutrient deficiency [4], is promoted as a particularly effective strategy to combat micronutrient deficiencies [5,6], though its effectiveness, risks, and ethical implications vary depending on the contexts associated with the deficiency it is addressing and the circumstances related to its implementation [7].

However, food fortification is just one among many nutrition interventions available to combat micronutrient deficiencies. These interventions are commonly considered as belonging to either of two broad categories:

(i) Nutrition-specific interventions (NSpI) “address the immediate causes of undernutrition, like inadequate dietary intake, and some of the underlying causes, like feeding practices and access to food” [8]. Food fortification and nutrient supplementation are prominent examples of these interventions that can rapidly increase an individual’s and targeted population’s exposure to a nutrient(s) [9].
(ii) Nutrition-sensitive interventions (NSeI) “can address some of the underlying and basic causes of malnutrition by incorporating nutrition goals and actions from a wide range of sectors. They can also serve as delivery platforms for nutrition-specific interventions” [8]. Interventions may be located in sectors as diverse as agriculture [10], education [11], and social support [12]. In contrast to nutrient-oriented interventions, they generally focus on promoting food and diet quality and diversity to support consumption of a healthy, balanced diet.

Whether or not to select food fortification as an intervention to combat a micronutrient deficiency will depend largely on the circumstances associated with this nutrition problem and what alternative interventions are available. In the short term, a NSpI such as food fortification may be well suited to treat an acute micronutrient deficiency. Alternatively, in a chronic situation, food-based NSeI are likely to be better suited to protect against and sustainably prevent micronutrient deficiencies. Over the longer term, food- and diet-based interventions are better able to tackle the co-existence of multiple micronutrient deficiencies, and deliver social and economic co-benefits [13]. Generally, a strategically implemented combination of multiple interventions is recommended as the best approach to achieve nutrition’s full potential for health and development outcomes [8,14,15,16]. As Uauy comments, “Fortification should be seen as complementary to food-based strategies and not as a replacement to dietary diversification, and can serve as a cost-effective measure to resolve micronutrient deficiencies until food-based approaches become feasible” [17].

Evidence-informed policy and practice decisions are required to plan and implement nutrition policies and interventions. The WHO “Handbook for Guideline Development” [18] explains that the WHO guideline recommendations need to be based on the best evidence available and that an effective approach for obtaining such evidence is to draw upon the findings of systematic reviews (SRs) of relevant interventions. A number of relevant and detailed SRs, guideline statements, and implementation actions to combat micronutrient deficiencies are available. Rationally, it would be expected that SRs would assess all available evidence for both NSpI and NSeI. In turn, these reviews would inform the development NSpI- or NSeI-aligned guideline statements and implementation actions. However, food policy-making is an inherently political process, especially given the contested views over the causes of, and solutions to, complex problems such as micronutrient deficiencies [7]. For example, political processes may influence what evidence is collected, synthesized, and translated in the formulation of policies and interventions to combat micronutrient deficiencies. Our hypothesis was that the various nutrition interventions available to help combat micronutrient deficiencies are not equally represented in SRs, that this will impact the number and type of guidelines and policy statements formed and, finally, that this will translate into a skewed number and type of implemented actions for nutrition. As far as we know, the profile of and relationships among these three sets of resources have not been investigated. Thus, the aim of this research was to analyse the profile of nutrition interventions for combating micronutrient deficiency with particular focus on food fortification reported in existing SRs, guidelines and policy statements, and implementation actions for nutrition. The findings from this research can help inform the future planning of evidence synthesis, guideline development, and implementation actions for nutrition interventions to combat micronutrient deficiencies.

## 2. Methods

Three forms of evidence synthesis and translation were analysed: Published SRs; nutrition guidelines and policy statements; and implementation of nutrition actions.

### 2.1. Selection of Records

We searched and selected records for inclusion as follows:

#### 2.1.1. Systematic Reviews

To identify relevant SRs and SR protocols, we conducted a rapid search of four databases, namely PubMed, Epistemonikos, TRIP (31 May 2016), and Health Evidence (3 June 2016). The Cochrane Database of Systematic Reviews (CDSR) had been previously screened for another study (31 July 2015) to identify nutrition-related reviews. We searched this database for reviews that met the inclusion criteria for this study. The search strategies employed for the first four listed databases are outlined in Appendix A. Two authors (SD and CN) screened titles and abstracts, and full-texts if necessary, to identify eligible studies. Disagreements were resolved through discussion.

Eligible studies were SRs and SR protocols of interventions that addressed any primary micronutrient deficiencies, either through fortification (including biofortification and targeted fortification) or other methods, in any population. Systematic reviews of nutrition interventions that addressed outcomes regarded as well-established manifestations of micronutrient deficiencies such as poor growth and adverse neurodevelopmental outcomes were also included. Systematic reviews were defined as those reviews that had predetermined objectives, had predetermined criteria for eligibility, searched for at least two data sources of which one needed to be an electronic database, and performed data extraction and risk of bias assessment [19].

#### 2.1.2. Nutrition Guidelines and Policy Statements

Data for nutrition guidelines and policy statements were collected from the WHO e-Library of Evidence for Nutrition Actions (eLENA) [20]. This database is an online library of nutrition interventions, categorised according to the level of evidence that supports them:

“Category 1 interventions are interventions for which there are guidelines that have been recently approved by the WHO Guidelines Review Committee (GRC). Category 1 interventions also include those supported by recommendations and other forms of guidance that have been adopted or endorsed by the World Health Assembly”

[21]

“Category 2 interventions are interventions for which systematic review(s) have been conducted but no recent guidelines are yet available that have been approved by the WHO Guidelines Review Committee”

[21]

“Category 3 interventions are interventions for which available evidence is limited and systematic reviews have not yet been conducted”

[21]

To identify relevant records, the complete list of interventions included in the eLENA was screened by title (and content if necessary) by one author (KW) and cross-checked by another author (ML). Disagreements were resolved through discussion. Interventions that sought to prevent or treat micronutrient deficiencies were eligible for inclusion. Interventions addressing macronutrient deficiencies were excluded, as were those addressing overweight/obesity or diet-related non-communicable diseases. Additionally excluded was mandatory folic acid fortification as an intervention to reduce the risk of neural tube defects (NTDs). The rationale for this exclusion was that the relationship between folic acid and NTD risk reduction is not conceptually equivalent to that associated with the causation of a conventional micronutrient deficiency disease. Although the precise mechanism by which folic acid exerts its protective influence is not known, NTD risk is reduced when a “dose” of folic acid, beyond that required to meet the folate recommended dietary intake level, is consumed by at-risk individuals, i.e., in this circumstance, folic acid is acting more as a therapeutic agent than as a conventional nutrient [7]. Interventions that sought to prevent anaemia through folic acid supplementation were eligible for inclusion.

#### 2.1.3. Implementation of Nutrition Actions

Data on the implementation of nutrition actions were collected by interrogating the WHO Global database on the Implementation of Nutrition Action (GINA) [22]. In this database, individual nutrition actions are classified under topics and sub-topics. They are also categorised as ‘ongoing’, ‘completed’, or ‘planned’. Our search was conducted in June 2016. Using the filter options available on the website, we limited our search to nutrition actions classified under each of the following topics: Micronutrient supplementation; food fortification; maternal, infant, and young child nutrition; nutrition and infectious disease; nutrition in schools; and nutrition sensitive actions. For the purposes of this project, it was neither necessary nor feasible to assess the large number of individual nutrition actions. Instead, eligibility criteria were applied at the sub-topic level. The same set of inclusion and exclusion criteria was applied to results within the eLENA and the GINA: Sub-topics that described actions to prevent or treat micronutrient deficiencies were eligible for inclusion; sub-topics that described actions to address macronutrient deficiency were excluded, as were those describing actions to address overweight/obesity or diet-related non-communicable diseases and those that involved mandatory folic acid fortification to reduce the risk of NTDs. Sub-topics were screened by title by one author (KW) and cross-checked by another author (ML). Disagreements were resolved through discussion.

### 2.2. Data Extraction and Analysis

#### 2.2.1. Systematic Reviews

We categorized interventions addressed in the included reviews as fortification, supplementation, or other interventions. For those that addressed fortification, we extracted information on whether fortification was with single micronutrients (i.e., specified up to three micronutrients) or with multiple micronutrients (i.e., more than three micronutrients), as well as the type of food vehicle used. We calculated the proportions of included SRs and SR protocols assessing food fortification, supplementation, and other types of interventions for micronutrient deficiencies, as well as proportions of reviews addressing food fortification with single or multiple micronutrients. Data were presented descriptively.

#### 2.2.2. Nutrition Guidelines and Policy Statements

We classified included interventions by intervention type (food fortification, micronutrient supplementation, or other) and by the level of evidence supporting them (Category 1, 2 or 3, as described above). We then calculated the number and proportion of interventions by type and by category. Interventions were also classified as NSpI or NSeI and described accordingly.

#### 2.2.3. Implementation of Nutrition Actions

Sub-topics that met the inclusion criteria were classified by the type of nutrition actions being implemented (food fortification, micronutrient supplementation, or other). The number and proportion of nutrition actions were then calculated. Actions were also classified as NSpI or NSeI and described accordingly.

## 3. Results

### 3.1. Systematic Reviews

**Figure 1:**
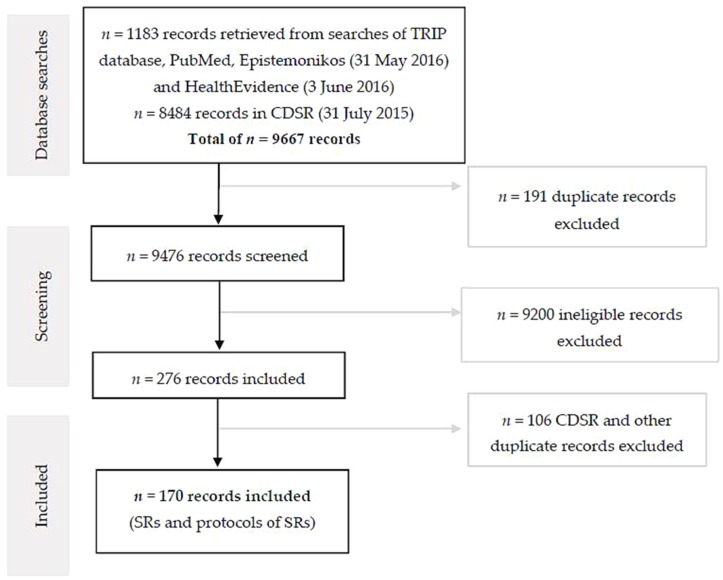
Flowchart of review selection process. CDSR: Cochrane Database of Systematic Reviews.

The review selection process for SRs and SR protocols is outlined in Figure 1. Searches of PubMed, TRIP, Epistemonikos, and Health Evidence retrieved 1183 records, of which 92 met our eligibility criteria (excluding records from the CDSR). In the CDSR, of 8484 records identified, 78 were included in this analysis. In total, 170 SRs and SR protocols (including one overview of SRs) were included that addressed primary micronutrient deficiencies, either through fortification or other methods, in any population.

Of the 170 included SRs and SR protocols, the majority addressed food fortification-only (*n* = 29, 17.0%) or micronutrient supplementation-only interventions (*n* = 108, 63.5%) (Table 1).

Of the 29 fortification-only interventions, 14 involved fortification with multiple micronutrients and 15 involved single micronutrients (iron *n* = 5, iodine *n* = 4, zinc *n* = 3, vitamin A *n* = 1, vitamin D *n* = 1, and calcium and vitamin D *n* = 1). Vehicles for fortification included staple foods, maize flour, wheat flour, rice, salt, condiments, seasonings and various foods (e.g., milk, margarine for vitamin D, and point-of-use fortification of foods with micronutrient powders), as well as dairy and non-dairy beverages. The vast majority of the supplementation-only interventions involved single nutrients (*n* = 92) rather than multiple micronutrients (*n* = 16).

Some SRs and SR protocols addressed other interventions (*n* = 12, 7.0%) or a combined intervention (*n* = 21, 12.3%). Other interventions included food-based interventions (e.g., school feeding programs, dietary diversification, and modification strategies), targeted financial incentives (e.g., conditional cash transfer programs), agricultural interventions (e.g., household food production strategies), and education-based interventions. Combined interventions included fortification and supplementation (*n* = 6), fortification, supplementation and other interventions (*n* = 7), supplementation and other interventions (*n* = 3), and fortification and other interventions (*n* = 5).

All of the fortification-only and supplementation-only interventions were NSpI. More than half of the ‘other’ interventions addressed by SRs were NSpI, such as behaviour change interventions to promote improved breastfeeding and complimentary feeding, and food-based feeding program interventions. The remainder was made up of NSeI, such as those using agricultural or social security platforms.

### 3.2. Nutrition Guidelines and Policy Statements

69 of the 101 interventions in the eLENA met our inclusion criteria (Table 2). Food fortification interventions included the fortification of staple foods such as flour and rice, iodisation of salt, and the use of multiple micronutrient powders for the fortification of foods in the home. One intervention involved biofortification of staple crops with iron, zinc, or vitamin A. Supplementation interventions included vitamins A, D, and E, zinc, iron, folic acid (in combination with zinc or iron), and calcium.

All interventions involving supplementation were classified as Category 1 or 2 interventions (*n* = 34). Of the fortification interventions, four of seven were Category 1 or 2 interventions, and three of seven were Category 3 interventions. A number of other relevant interventions were also identified. Other interventions included those addressing feeding practices, marketing of infant formula, nutrition assessment, nutrition education, sanitation, and financial incentives. All other interventions were classified as either Category 1 or Category 2 interventions (*n* = 28).

Of the 69 included interventions, the vast majority (*n* = 63, 91.3%) were NSpI. The remaining six interventions (8.7%) were NSeI (agricultural interventions *n* = 1, public health interventions *n* = 4, social welfare interventions *n* = 1). Five of the six NSeI interventions were classified as Category 1 or Category 2 interventions.

### 3.3. Implementation of Nutrition Actions

Thirty sub-topics in the WHO GINA database were included in our analysis. A total of 1646 nutrition actions were categorised under these sub-topics (Table 3).

Some nutrition actions involved food fortification (*n* = 293, 17.8%) or micronutrient supplementation (*n* = 438, 26.7%), but more than half did not (*n* = 915, 55.6%). Vehicles for food fortification included rice, maize or corn flour, wheat flour, milk, condiments and seasonings, sugar, margarine, butter, and oil. Some actions involved multiple micronutrients, such as fortification of salt with iron, iodine, vitamin B12, folic acid, zinc, and vitamin A. A number of nutrition actions involved the addition of multiple micronutrient powders to foods in the home. Nutrition actions involving micronutrient supplementation involved single nutrients (zinc, iodine, iron and folic acid, iron, vitamin A, and calcium) or multiple micronutrients. Other nutrition actions included both NSpI and NSeI. Overall, the majority of actions were NSpI (*n* = 1169, 71.0%). The remaining 477 actions were NSeI (agricultural actions *n* = 22, public health actions *n* = 439, and other nutrition sensitive actions *n* = 16).

## 4. Discussion

The findings from this analysis indicate that there is a substantial body of evidence reporting the use of food fortification as an intervention to combat micronutrient deficiencies. They also indicate that this evidence is translating into practice to a certain extent. A range of food fortification interventions are addressed by the SRs, guidelines and policy statements, and nutrition actions. All three forms of evidence synthesis and translation include interventions to combat vitamin A, iodine, iron, and zinc deficiencies noted with particular attention at ICN2 [1].

Micronutrient supplementation is the dominant intervention type addressed in included SRs, protocols, guidelines, and policy statements. Systematic reviews and protocols related to NSpI, which are predominantly supplementation and fortification, are significantly more available than those related to NSeI alone or in combination with NSpI. The fact that there are significantly more guidelines, policy statements, and actions available related to NSpI than to NSeI alone or in combination with NSpI confirms our original hypothesis. These findings are consistent with a rational linear relationship pattern connecting the evidence synthesized from SRs, the development of guideline statements, and implementation actions, at least in relation to NSpI. However, further studies would be needed to confirm this.

The dominance of NSpI-related reviews and protocols, guidelines and policy statements, and actions relative to NSeI is not congruent with public health best practice, including recommendations by the United Nations International Children's Emergency Fund (UNICEF) [8] and the WHO [14], which encourage a strategic combination of evidence-informed interventions to combat micronutrient deficiencies. Consequently, policies and practices might not be as effective as they might be and may result in public health risks. Whereas NSpI are well suited to relatively quickly treat and prevent the physiological symptoms of micronutrient deficiencies, they may not address the underlying cause of the deficiency as NSeI would. Consequently, depending on the circumstances, NSpI may not be as effective, sustainable, or safe as NSeI [7]. Concerns about risks associated with the potential for harmful dietary exposure resulting from food fortification interventions have been raised in the context of low- to middle-income countries [23] as well as high-income countries [24,25].

It was beyond the scope of this research to investigate specific explanations for the observed dominance of NSpI in general, and supplementation and fortification interventions in particular, among the interventions available to combat micronutrient deficiencies. However, the literature does provide examples where methodological orthodoxies and political factors can influence decision-making and result in the privileging of supplementation and food fortification interventions over NSeI. Methodologically, evidence synthesis orthodoxies typically rate the evidence obtained from studies using reductionist methods higher than evidence obtained from studies using more holistic methods. Investigations of relationships between single nutrients and single physiological outcomes are more amenable to reductionist methods than the inherently complex relationships that exist between NSeI and health outcomes [26,27]. Politically, vested interests can preferentially support NSpI relative to NSeI, and this manifests in relatively greater likelihood that certain research agendas are pursued, certain research questions are posed, certain SRs are conducted, and certain interventions are implemented. For example, potential conflicts of interest that can arise with the planning of food fortification activities were identified in the WHO technical report, “Addressing and managing conflicts of interest in the planning and delivery of nutrition programmes at country level” [28]. In the report, it was noted: “The private sector influences the political framing of the problem and the selection of appropriate policy responses (at international and national level) in the following areas: Reliance on private money; research investment and prioritization; underlying assumptions; selection of experts, advisers, and researchers; partnerships or donors pushing for fortification when it is not needed; de-prioritization of medium- and long-term solutions … (and at-risk areas include inadequacies when) … evaluating options (e.g., fortification versus other nutrition interventions)” [28] (p. 22).

The strengths of this research included the novel nature of the investigation, the high quality databases from which the data were sourced and the critical analysis of the collective profile of these data, their inter-relationships, potential knowledge gaps, implications for public health nutrition practice, and future research priorities. Nonetheless, the potential for limitations with the collection and analysis of the data cannot be discounted. Comprehensive data on systematic reviews were collected from five key databases; however, it is possible that we missed relevant SRs. Similarly, we may have missed relevant nutrition guidelines and policy statements and actions that are being implemented but are not recorded in the eLENA and the GINA, respectively. In the GINA the data extraction was restricted to actions only and not policies. It is possible that, if policies had also been included, the findings may have differed to a certain extent. The decision not to include policies was based on the authors’ experience that the existence of a policy does not always lead to an action, and it was action that was the priority interest for this investigation. Additionally, in relation to the GINA, the search relied on the classifications in the website database rather than on original keyword search, so relevant results may have been missed. Despite quality assurance procedures being undertaken, it is possible there were inaccuracies regarding data extraction, coding, and interpretation during the data analysis component of the research. We did not assess the quality of individual reviews, guidelines, or actions included in the study, as this would have been beyond the scope of the research aim. We also did not investigate the reach and impact of individual interventions. Therefore, a food fortification intervention to modify the nutrient composition of the national food supply and an NSeI such as a local agriculture program were recorded as one intervention each, despite the reality that the former would have a significantly higher reach and impact.

To the authors’ knowledge this is the first study to investigate SRs, guidelines and policy statements, and actions by type of nutrition intervention to combat micronutrient deficiencies, as well as analyse the relationship between these three activities. A number of unanswered questions remain. Priority areas for future research include investigating the relationship between evidence synthesized for NSeI and what then is happening in policy and practice, especially if this can contribute to the informing of strategic combinations of interventions to combat micronutrient deficiencies; extending the investigation to analyse policy documents in the GINA; and investigating how and why there is a dominance of NSpI over NSeI in the observed SRs, guidelines and policy statements, and actions observed.

## 5. Conclusions

Food fortification can be a powerful intervention for helping combat micronutrient deficiencies, especially when planned and implemented as a component of a strategic combination of interventions. Currently, there is evidence available for a range of food fortification interventions, and there has been much translation into action. However, food fortification and especially micronutrient supplementation interventions are dominating what evidence is being synthesised, which may subsequently determine what guidelines and policy statements are being produced. Effective and safe policies and actions to combat micronutrient deficiencies will require decisions to be informed by a body of evidence on a variety of interventions. This does not necessarily mean less investment in NSpI but rather more investment in prioritizing and proactively conducting and making available SRs of alternative interventions that go beyond linear relationships between nutrients and health outcomes, and extending to mid-stream and upstream interventions, such as those addressing socio-ecological and environmental determinants. An increased variety in the types of interventions for which evidence synthesis is available would help inform a greater diversity of guidelines and policy statements and actions to counter the current dominance of supplementation and fortification interventions and increase the likelihood of comprehensive, sustainable, effective, and safe policies and actions to combat micronutrient deficiencies.

## Figures and Tables

**Table 1:** Number of systematic reviews and systematic review protocols addressing micronutrient deficiencies by type of intervention.

Type of Intervention	Number of Records
Fortification only	29 (17.0%)
Supplementation only	108 (63.5%)
Other interventions	12 (7.0%)
Combined interventions	21 (12.3%)
Total	170

**Table 2:** Number of nutrition interventions in the e-Library of Evidence for Nutrition Actions (eLENA) addressing micronutrient deficiencies, by type of intervention and level of supporting evidence.

Level of Supporting Evidence	Type of Intervention			
	Fortification	Supplementation	Other Interventions	Total
Category 1 intervention	3	21	13	37
Category 2 intervention	1	13	15	29
Category 3 intervention	3	0	0	3
Total	7	34	28	69

**Table 3:** Number of nutrition actions in the Global database on the Implementation of Nutrition Action (GINA) addressing micronutrient deficiencies, by type of action.

	Number of Actions	Nutrition Specific Actions	Nutrition Sensitive Actions
Fortification	293	292	1
Supplementation	438	438	0
Other nutrition actions	915	439	476
Total	1646	1169	477

## Appendix A

**Table A1:** Search strategy for systematic reviews and systematic review protocols.

Database	Search Query	
PubMed	#1	(Vitamin*[tiab] OR mineral*[tiab] OR nutrient*[tiab] OR micronutrient*[tiab]) AND (deficiency[tiab] OR deficiencies[tiab] OR malnutrition[tiab] OR undernutrition[tiab])
	#2	“systematic review”[tiab] OR meta-analysis[pt] OR meta-analysis[tiab]
	#3	#1 AND #2
Epistemonikos	((title: ((Vitamin* OR mineral* OR nutrient* OR micronutrient*) AND (deficiency OR deficiencies OR malnutrition OR undernutrition)) OR abstract: ((Vitamin* OR mineral* OR nutrient* OR micronutrient*) AND (deficiency OR deficiencies OR malnutrition OR undernutrition))), by publication type: systematic reviews	
Health Evidence	((Vitamin OR vitamins OR mineral OR minerals OR nutrient OR nutrients OR micronutrient OR micronutrients) AND (deficiency OR deficiencies OR malnutrition OR undernutrition))	
TRIP	(Vitamin OR vitamins OR mineral OR minerals OR nutrient OR nutrients OR micronutrient OR micronutrients) AND (deficiency OR deficiencies OR malnutrition OR undernutrition)	
CDSR	Entire database screened previously for another study	
CDSR: Cochrane Database of Systematic Reviews		
